# Comparing the Prognoses of Breast-Conserving Surgeries for Differently Aged Women with Early Stage Breast Cancer: Use of a Propensity Score Method

**DOI:** 10.1155/2022/1801717

**Published:** 2022-04-23

**Authors:** Shurui Bao, Guijin He

**Affiliations:** Department of Oncology, Shengjing Hospital of China Medical University, Shenyang 110013, Liaoning, China

## Abstract

**Background:**

To explore the effect of age on the prognosis of patients with early stage breast cancer after breast-conserving surgery (BCS) and to provide references for young patients.

**Methods:**

All clinical data of patients with early breast cancer undergoing BCS who were treated at Shengjing Hospital of China Medical University from January 2011 to May 2016 were obtained. The primary endpoints were local recurrence (LR) and distant recurrence, and the secondary endpoint was breast cancer-specific survival (BCSS). Chi-squared tests and Fisher's exact tests were used for statistical analysis. Disease-free survival (DFS) and BCSS were calculated by Kaplan–Meier survival analysis and compared using log-rank tests. Logistic regression was used for multivariable analysis of the effect of age in different subgroups. Propensity score matching (PSM) was used to reduce the bias confounding factors on oncological outcomes.

**Results:**

Younger patients had higher Ki-67 expression (*P*=0.048) and larger tumors (*P*=0.042) compared to older patients. No other clinical features were significantly different between age groups. There was no significant difference between the two groups in BCSS (*P*=0.186); however, DFS was significantly different before PSM (*P*=0.012). Triple-negative breast cancer and Ki-67 positivity combined with younger age at diagnosis were associated with a higher risk of recurrence (*P*=0.018 and *P*=0.046, respectively). After PSM, there were no significant differences in BCSS nor DFS between the two age groups (*P*=0.559 and *P*=0.261, respectively).

**Conclusion:**

BCS for young patients is not associated with increased DFS nor BCSS. However, young patients with triple-negative breast cancer and/or Ki-67 positivity have a poor prognosis. In sum, BCS may be appropriate for a subgroup of young patients.

## 1. Background

Breast cancer is one of the most common malignancies worldwide and one of the leading causes of death in women. Unlike Western countries, as high as 20% of patients in Asian countries with breast cancer are young. Standard treatment options are breast-conserving surgery (BCS) with adjuvant radiotherapy, which result in better cosmetic outcomes and improve patient satisfaction and quality of life [1–4]. However, the safety of BCS in young women with early stage breast cancer is controversial. Some retrospective studies suggest that the risk of local recurrence is higher in young patients with breast cancer treated with BCS compared to older patients [5–8]. Breast cancer tends to be more aggressive in young patients, and young age is a well-known prognostic and predictive factor that can affect disease-free survival (DFS) and overall survival [9–11]. Epidemiology shows that approximately 6.5% of women diagnosed with breast cancer are ≤40-year-old and are defined as young breast cancer patients [7, 12]. However, whether age is an independent prognostic factor remains controversial [13, 14]. Younger women with breast cancer tend to have worse pathological staging, and therefore, the worse outcomes observed in young patients are often attributed to these unfavorable clinicopathologic features [15, 16]. In China, we lack long-term follow-up reports of young women after BCS for breast cancer treatment. Through this retrospective cohort study, we examined the effect of age on survival, using 40 years as the cutoff age. We attempted to compare the outcomes of younger and older patients with early breast cancer undergoing BCS who were treated consecutively at a single center.

## 2. Methods

### 2.1. Patient Selection

**Figure 1:**
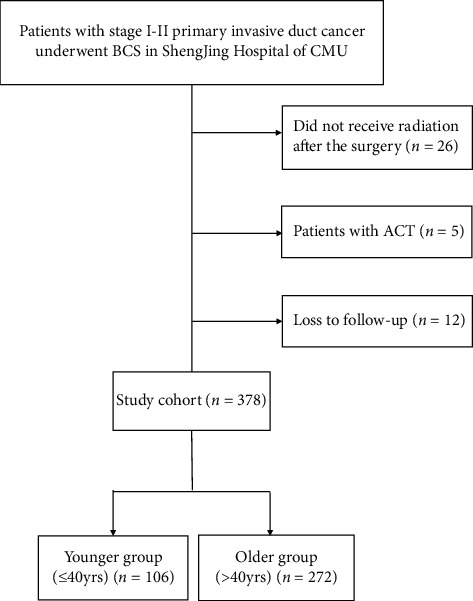
Flowchart of selection.

We retrieved clinical data of patients undergoing BCS for early breast cancer treated in Shengjing Hospital of China Medical University (CMU) from January 2011 to May 2016. Our study was submitted to and approved by the Ethics Committee of Shengjing Hospital of CMU. We made phone calls to patients or their families to obtain follow-up information. Follow-up started from the surgery to May 2021 or death of the patient. The inclusion criteria were as follows: no contraindications to BCS and receipt of BCS, stages I-II primary invasive breast cancer, unilateral breast cancer, and receipt of radiation after surgery. We also had the following exclusion criteria: inflammatory breast cancer, male breast cancer, presence of other malignant tumors or serious illnesses, and incomplete data. Of all the cases, 12 were lost to follow-up, 31 were excluded, and 378 cases were eventually included. The selection process for patients is shown in Figure 1.

### 2.2. Treatment and Follow-Up

BCS consists of wide local excision of the tumor with appropriate axillary node management followed by adjuvant whole-breast radiation therapy and systemic treatment if necessary. We confirmed that the pathological diagnosis of the resection margins of all patients was negative through both frozen and permanent biopsy. We have excluded patients who did not undergo radiotherapy after BCS. Patients received adjuvant therapy according to their preference and the National Comprehensive Cancer Network (NCCN) guidelines. A cutoff ≥14% staining was used to indicate Ki-67 positivity. For ERs and PRs, ≥1% nuclear expression was considered positive. All patients were classified into three subtypes: HR+ (ER/PR+, HER2−), HER2+, and TNBC (ER−, PR−, HER2−). For the first 5 years after surgery, all patients received bilateral breast and axillary ultrasound every 6 months and bilateral mammography, chest X-ray, and ultrasounds of the liver, gallbladder, and spleen once a year. The endpoints of our study were DFS and breast cancer-specific survival (BCSS). DFS is defined as the time interval from surgery to the appearance of any local recurrence or distant metastasis or breast cancer progression leading to death. BCSS is defined as time interval from surgery to breast cancer progression leading to death.

### 2.3. Statistical Analysis

All statistical analyses were performed using the SPSS 26.0 software. All tests were two-sided with a level of significance set at 5%. The clinical-pathological characteristics of the younger and older patient groups were analysed as categorical variables and compared by chi-squared tests and Fisher's exact tests. DFS and BCSS were calculated by Kaplan–Meier survival analysis and compared using log-rank tests. To reduce the bias of confounding factors on oncological outcomes, we used propensity score matching (PSM). The following covariates were included into our model to balance between the 2 groups: *T* stage, LN status, Ki-67 status, molecular subtypes, adjuvant chemotherapy, and adjuvant endocrine therapy status. We set the caliper value as o, and cases were 1 : 1 matched into two groups without replacement. Eventually, we matched 90 pairs in both groups.

## 3. Results

### 3.1. Clinical Features

Our cohort included a total of 272 patients in the older group (>40 y) and 106 patients in the younger group (≤40 y). Their clinical characteristics are given in Table 1. Before PSM, we found that the younger group had a higher rate of Ki-67 positivity (*P*=0.048) and larger average tumor size (*P*=0.042) than the older group. No other clinical features were significantly different between the age groups. However, the younger group was more likely to receive adjuvant chemotherapy.

### 3.2. Prognosis

**Figure 2:**
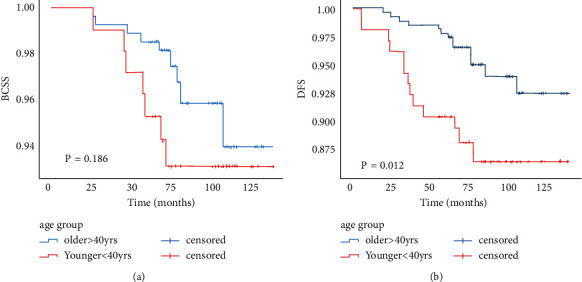
(a) BCSS by age before PSM. (b) DFS by age before PSM.

Before PSM, the median follow-up period for the cohort was 78 months (range, 26–140 months). During the follow-up, there were 9 patients who died due to breast cancer-related disease in the older group and seven patients in the younger group. Kaplan–Meier survival curves showed no significant difference between the two age groups in BCSS (P0.186); however, DFS was significantly different (*P*=0.012) (Figure 2). There were 5 cases local recurrence, 11 cases distant recurrence, and 3 cases with both in the younger group. There were 6 cases of local recurrence, 11 cases of distant recurrence, and 4 cases with both in the older group. The main difference in DFS came from distant metastasis (Table 2). When we stratified by factors, the logistic models showed the younger group was more prone to recurrence compared to the older group when comparing patients positive for Ki-67 status and TNBC (OR = 3.9, *P*=0.018; OR = 5.833, *P*=0.046); however, this was not true for other subgroups.

**Figure 3:**
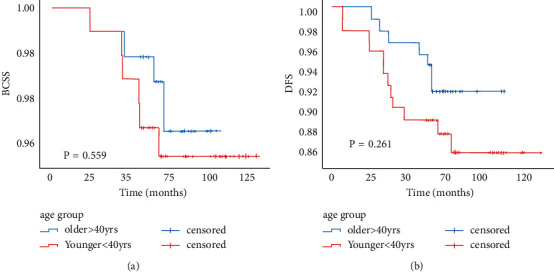
(a) Overall survival (OS) by age after PSM. (b) Disease-free survival (DFS) by age after PSM.

After PSM, we had 90 pairs for both groups. The median follow-up periods were 108 months (range, 25–114 months) in the older group and 125 months (range, 6–114 months) in the younger group. There were 7 instances of recurrence in the older group recurrence, including 4 cases of local recurrence and 4 cases of distant recurrence. The younger group had 12 cases of recurrence in the younger group, including 7 cases of local recurrence and 7 cases of distant recurrence. Kaplan–Meier survival curves showed that the younger group tended to have a worse prognosis; however, log-rank tests showed no significant differences in BCSS or DFS between two groups (Figure 3). We then used a paired *χ*^2^ test (McNemarʼs test) to compare the matched pairs within 5 years, and age had no effect on oncology outcomes (Table 3).

## 4. Discussion

To our knowledge, there are few matched cohort analyses that compare oncology outcomes of younger (≤40-year-old) and older patients undergoing breast conservation surgery in China. Our study only focused on cases of the most common early invasive ductal carcinoma of early stage breast cancer in our department. Because of the major effect of radiotherapy on postoperative recurrence as described by the NCCN guidelines, we first excluded patients who did not undergo postoperative radiotherapy. We also excluded patients who underwent BCS with neoadjuvant chemotherapy (NACT) in the past decade, given the small number of patients and the controversial definition of margins. The data of patients who did not undergo adjuvant chemotherapy were used to elucidate the impact of age on prognosis. For the total cohort, the five-year BCSS and DFS were 97.6% and 96.0%, respectively, which are impressive oncology outcomes.

Previous literature has shown that young age is a well-known risk factor for poor oncology outcomes, especially higher recurrence rates [17, 18]. Although this may due to many unfavorable prognostic factors, BCS is still controversial for younger patients. Our major finding is that age itself is not an independent prognostic risk factor after BCS; however, the combination of young age with other clinical features was associated with higher risk of recurrence. Compared with older women, the younger group had fewer luminal and Her2-positive patients and more TN patients. We found the proportions of patients with T2 stage and Ki-67 positivity were higher in the younger group. These features may be due to differences in the choice of surgery for patients of different ages. Young patients tended to choose BCS even if their tumors are large and their pathological types are aggressive.

We found there is no significant increase in the risk of BCSS in the younger patients, but DFS in the younger group was significantly worse before PSM. We further divided recurrence into local recurrence and distant metastasis and found that the difference mainly originated from distant recurrence, consistent with previous literature [5, 8]. However, the risk of local recurrence in the young group undergoing BCS was not different compared to the older group. This may be related to the improvement of breast conservation and decreasing the risk of local recurrence by local radiotherapy, which does not reduce the risk of distant metastasis [19, 20]. When further stratified by confounding factors, young women with Ki-67 positivity and TN tumors tended to have higher rates of recurrence than older counterparts, while age did not have impact on recurrence in other subgroups. The outcomes indicated that younger patients with Ki-67 positivity and/or TN tumors should be more cautious in choosing BCS. Next, we used PSM to eliminate the influence of covariates and found no significant differences between the two age groups in BCSS and DFS. The results indicated that young patients had a worse prognosis after undergoing BCS that mainly depended on aggressive clinical characteristics. In total, our study suggests that there is no difference in BCSS of different age groups after BCS; however, the risk of recurrence after surgery may be higher, which requires analysis that combines clinical features and pathological results.

In comparison to Western countries, which have much lower rates of breast cancer cases in young women, 20% of new breast cancer cases in Asia are in patients under the age of 40. In China, there has been a lack of comparison of oncology outcomes stratified by age. Although a minority breast cancer cases occur in young women, these cases are associated with a range of essential effects. Oncology outcomes as well as physical and psychological outcomes may affect patients' attitudes toward treatment. Compared to the older patients, BCS instead of modified radical mastectomy may have longer and obvious psychological and social impacts on young people. Therefore, it is very important to designate an individualized treatment plan for young patients with breast cancer. Although there was no significant difference in oncology outcomes between the two groups after matching, BCS still needs to be carefully considered for young patients with Ki-67 positivity and TN tumors. Our study only illustrated the impact of Ki-67 status on age when 14% staining was used as the positivity threshold; however, it is still necessary to further determine the specific upper limit that is safe for young patients. Our findings may be limited by the facts that this was a retrospective single-institution study and that systemic treatments have improved during the follow-up periods.

## 5. Conclusion

Young women (≤40 years) who undergo BCS have a worse prognosis than old women, especially distant recurrence due to their aggressive clinical features. Young patients with Ki-67 positivity have a higher recurrence rate compared to older counterparts. Age was not an independent prognostic factor in our study, but it should be taken into consideration to decide the most reasonable treatment.

## Figures and Tables

**Table 1:** Clinical characteristics compared by age and before and after PS.

Characteristics before PSM	Younger group	Older group	*P*	Characteristics after PSM	Younger group	Older group	*P*
	*n* = 106	*n* = 272			*n* = 90	*n* = 90	
Subtypes				Subtypes			
HR+	73 (68.9%)	189 (69.5%)		HR+	66 (73.3%)	66 (73.3%)	
Her+	10 (9.4%)	39 (14.2%)		Her+	6 (6.7%)	6 (6.7%)	
TNBC	23 (21.7%)	42 (16.2%)	0.225	TNBC	18 (20.0%)	18 (20.0%)	1
T stage				T stage			
T1	90 (84.9%)	250 (91.9%)		T1	80 (88.9%)	80 (88.9%)	
T2	16 (15.1%)	22 (8.1%)	0.042	T2	10 (11.1%)	10 (11.1%)	1
Axillary lymph node metastasis				Axillary lymph node metastasis			
No	86 (81.1%)	220 (80.9%)		No	76 (84.4%)	76 (84.4%)	
Yes	20 (18.9%)	52 (19.1%)	0.956	Yes	14 (15.6%)	14 (15.6%)	1
Ki-67				Ki-67			
>14%	65 (61.3%)	136 (50%)		>14%	57 (63.3%)	57 (63.3%)	
≤63.	41 (38.7%)	136 (50%)	0.048	≤048	33 (36.7%)	33 (37.1%)	1
Adjuvant endocrine therapy				Adjuvant endocrine therapy			
No	34 (32.1%)	77 (29.6%)		No	25 (27.8%)	25 (27.8%)	
Yes	72 (69.7%)	195 (71.7%)	0.470	Yes	65 (72.2%)	65 (72.2%)	1
Adjuvant chemotherapy				Adjuvant chemotherapy			
No	7 (6.6%)	28 (10.3%)		No	2 (2.2%)	2 (2.2%)	
Yes	99 (93.4%)	244 (89.7%)	0.266	Yes	88 (97.8%)	88 (97.8%)	1

**Table 2:** Local recurrence and distant metastasis recurrence compared by age.

DFS		BCSS	
Older group recurrence within 5 years	4 (4.4%)	Older group death within 5 years	2 (2.2%)
Younger group recurrences within 5 years	8 (11.1%)	Younger group death within 5 years	5 (5.6%)
*P*	0.386		0.450

**Table 3:** Oncology prognosis within 5 year, using the matched pairs (*n* = 90) as the sampling units (McNemarʼs test).

	≤40 y	>40 y	*P*
All recurrence	13	13	0.012
Local recurrence	5	6	0.192
Distant recurrence	11	11	0.022

## Data Availability

The data generated or analysed during this study are included within the article.

## Abbreviations

CMU: China medical university
LR: Local recurrence
BCSS: Breast cancer-specific survival
PSM: Propensity score matching
NACT: Neoadjuvant chemotherapy
DFS: Disease-free survival
BCS: Breast-conserving surgery.
